# Mind the Gap: Adult Paradigms and Pediatric Realities in Blunt Traumatic Aortic Injury

**DOI:** 10.1016/j.jaccas.2026.106948

**Published:** 2026-03-11

**Authors:** Matylda M. Mazur, Anna Kędziora

**Affiliations:** a Kaufman Center for Heart Failure Treatment and Recovery, Heart Vascular and Thoracic Institute, Cleveland Clinic, Cleveland, Ohio, USA; b Department of Cardiac Surgery and Heart Transplantation, Institute of Heart Diseases, Wroclaw Medical University, Wrocław, Poland

**Keywords:** aorta, cardiac magnetic resonance, cardiovascular disease, computed tomography, hemodynamics, pediatric surgery, perfusion, thoracic, three-dimensional imaging, treatment

**Figure:**
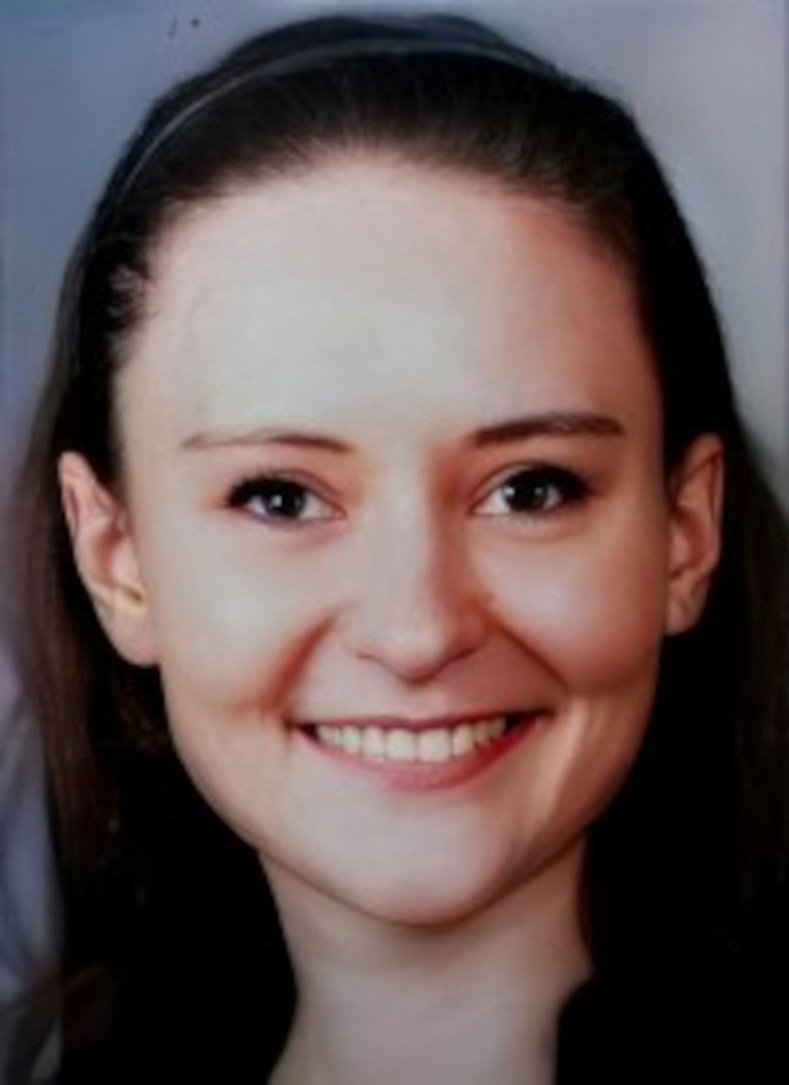

Aortic injuries in pediatric survivors of motor vehicle accidents are rare. However, the growing use of off-road and youth-sized all-terrain vehicles may shift this landscape, making these injuries an increasingly more common scenario observed in clinical practice. Management is challenging not only because these cases often involve complex, multiorgan trauma, but more so due to the absence of evidence-based guidelines, particularly regarding the timing and modality of intervention in pediatric patients. In this context, well-documented case reports are indispensable, as they illustrate real-world decision-making in high-stakes settings.

In this issue, Portuondo et al^1^ present a case of an 11-year-old who, following an all-terrain vehicle accident, sustained a transverse aortic arch pseudoaneurysm along with an esophageal tear and pulmonary and cardiac contusions. The latter warranted a 5-day-long veno-arterial extracorporeal membrane oxygenation support and a balloon atrial septostomy to be performed. Subsequently, the multidisciplinary team elected for tight blood pressure control, serial imaging, and other injuries stabilization. Eight weeks after the initial accident, a surgical transverse aortic arch pseudoaneurysm repair with an oversized Dacron graft was performed. Throughout the hospital stay, the patient remained neurologically intact. At 11 months postdischarge, the echocardiography was reassuring, and the patient returned to normal activity level.

One of the key clinical dilemmas is whether blunt traumatic aortic injury (BTAI) should be treated immediately or managed in a staged fashion with initial conservative stabilization. In pediatric patients, treatment strategy by large is extrapolated from the adult guidelines. While a delayed (>24 hours) approach is allowed for contained injuries in adults, it rarely exceeds 72 hours.^2^, ^3^, ^4^, ^5^, ^6^ The growing number of pediatric case reports demonstrate that the timing of an intervention can be delayed even by a couple of weeks, with favorable outcomes observed up to 4.5 years after the intervention.^7^^,^^8^ In the case presented by Portuondo et al, the surgical intervention was delayed for 8 weeks, which resulted in a good medium-term outcome. That could potentially suggest that high elasticity and compliance of the aorta in pediatric patients can be more forgiving than in an adult, where trauma is superimposed on pre-existing atherosclerosis and calcifications, making the aorta more fragile and prone to rupture.

Beyond determining the optimal timing, selecting the mode of intervention remains a challenge (Figure 1). In adults with high-grade BTAI, thoracic endovascular aortic repair (TEVAR) is currently the gold standard, with open surgical repair reserved for patients with unfavorable anatomy.^2^^,^^3^^,^^6^ Notably, these Class I recommendations are supported by a low Level of Evidence (LOE: C).^2^^,^^3^ In children, the same recommendations exist largely in a vacuum of pediatric-specific data, device approvals, and the scarcity of long-term outcome evidence.

**Figure 1:**
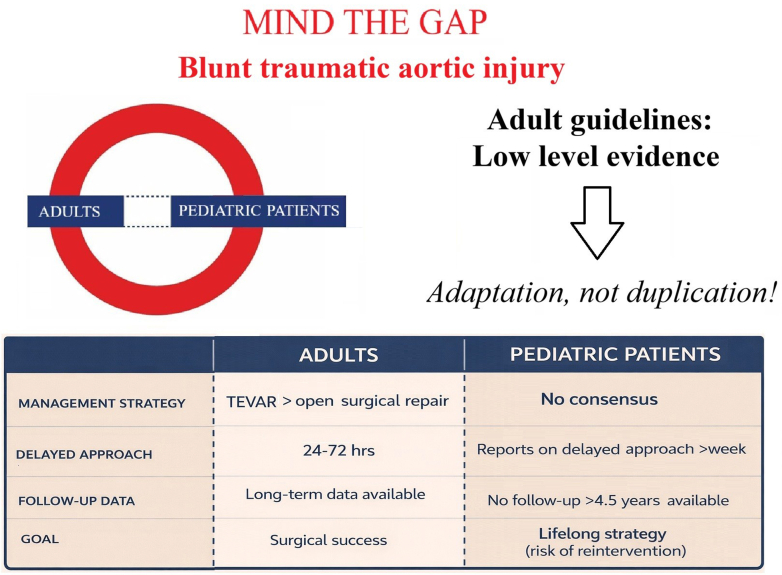
Challenges in Translating Adult Blunt Traumatic Aortic Injury Guidelines to Pediatric Practice

Adult literature overwhelmingly favors TEVAR, demonstrating lower early mortality and reduced rates of paraplegia and transfusion requirements.^9^ Meanwhile, pediatric patients undergoing TEVAR appear to have higher rates of traumatic brain injury than those undergoing open repair, without any difference in mortality.^8^ An increasing number of case reports support the use of TEVAR in children.^7^^,^^10^ The case presented in this issue was managed with open surgical repair. This approach should not be interpreted as a rejection of TEVAR. Rather, it reinforces that pediatric BTAI requires individualized decision-making, guided by lesion location, patient size, associated injuries, and institutional expertise. Multidisciplinary discussion is not ancillary here—it is central to safe and effective care.

The management of BTAI in children remains one of the most daunting surgical and cardiac challenges. The report by Portuondo et al underscores that the question is not simply whether to intervene, but *when* and *how*, and an individualized approach combined with the art of applying adult-derived algorithms may confer durable clinical success.

## Funding Support and Author Disclosures

The authors have reported that they have no relationships relevant to the contents of this paper to disclose.
